# Acidification reduced growth rate but not swimming speed of larval sea urchins

**DOI:** 10.1038/srep09764

**Published:** 2015-05-15

**Authors:** Kit Yu Karen Chan, Eliseba García, Sam Dupont

**Affiliations:** 1 Biology & Applied Ocean Physics and Engineering, Woods Hole Oceanographic Institution, Woods Hole, MA, U.S.A; 2 Division of Life Science, Hong Kong University of Science and Technology, Clear Water Bay, Hong Kong; 3 Departamento de Biología Animal (Ciencias Marinas), Universidad de La Laguna, La Laguna, Tenerife, Islas Canarias, Spain; 4 Department of Biological and Environmental Sciences, University of Gothenburg, The Sven Lovén Centre for Marine Sciences – Kristineberg, Fiskebäckskil, Sweden

## Abstract

Swimming behaviors of planktonic larvae impact dispersal and population dynamics of many benthic marine invertebrates. This key ecological function is modulated by larval development dynamics, biomechanics of the resulting morphology, and behavioral choices. Studies on ocean acidification effects on larval stages have yet to address this important interaction between development and swimming under environmentally-relevant flow conditions. Our video motion analysis revealed that pH covering present and future natural variability (pH 8.0, 7.6 and 7.2) did not affect age-specific swimming of larval green urchin S*trongylocentrotus droebachiensis* in still water nor in shear, despite acidified individuals being significantly smaller in size (reduced growth rate). This maintenance of speed and stability in shear was accompanied by an overall change in size-corrected shape, implying changes in swimming biomechanics. Our observations highlight strong evolutionary pressure to maintain swimming in a varying environment and the plasticity in larval responses to environmental change.

Many marine benthic organisms have limited mobility as adults and rely on a planktonic larval phase for dispersal. Planktonic larvae thus play a significant role in determining population abundance, genetic diversity, distribution, and their resilience to disturbances^1^,^2^. Larval survival, transport, and successful settlement are affected by environmental variables (e.g., prey and predator concentrations, current direction and speed) which is in turn modulated by larval swimming behaviors^3^,^4^,^5^. Larval swimming is bounded by the physiological and biomechanical limits of the larval form, and modulated through behavioral choices^6^,^7^,^8^.

Global stressors such as ocean acidification (OA) can affect larval growth and development. These stressors can lead to changes in organisms' morphology^9^ and modify dynamics of development^10^. Larval ability to carry out ecological functions are tightly coupled with their development and morphology. For example, changes in length and orientation of the ciliated arms of larval urchins affect their abilities to filter particles for food^11^, swim in still water^12^,^13^, and maintain directed movement (stability) in flow^14^. This tight coupling suggests that any changes associated with environmental stressors can have substantial consequences.

To date, very few studies have investigated how observed effects of OA on larval development and morphology can impact swimming behavior, and thus, potential consequences for successful dispersal. Non-invasive video motion analysis has been applied to investigate swimming of larval sand dollars *Dendraster excentricus* in still water^15^. Their results suggested that OA condition did not affect larval swimming speeds. The authors hypothesized that a coordinated change in shape could help maintain swimming performance. Planktonic larvae, however, are rarely found in completely still water in nature. They rather experience moving water, and thus, it is important to explore larval swimming performance in flow^16^,^17^.

The aim of this paper was to test the effect of OA on the relationship between developmental dynamics, larval morphology (with an emphasis on overall shape using geometric morphometrics, Fig. 1d), and swimming behavior (in still and moving water using non-invasive video motion analysis, Fig. 2). We exposed our focal organism, the larval green urchin *Strongylocentrotus droebachiensis*, to different rearing pHs (see Suppl. Table 1 for detailed carbonate chemistry) and compared their overall shapes and swimming behaviors in still water and in shear over time.

## 

**pH affected growth but not mortality rate**

Relative mortality rate (RMR in day^−1^) was calculated as the coefficient of the significant linear regression between relative density and time (days post fertilization). All regressions were statistically significant (p > 0.001) and had r^2^ values ranged from 0.59 to 0.85 (Suppl. Table 2). RMR were 0.036±0.0045 day^−1^ for pH 8.0, 0.039±0.0043 day^−1^ for pH 7.6, and 0.027±0.0097 day^−1^ for pH 7.2. Neither pH nor replicate had a significant effect on larval mortality (p = 0.21 and p = 0.28 respectively, Fig. 3a).

A logarithmic regression between total body length (mm) and days post-fertilization (hereafter larval age) was used to determine larval growth rate. The coefficient of regression between body length and the natural logarithm of larval age denotes the growth rate in a unit of mm ln (day)^−1^. All regressions were statistically significant (p > 0.001) and had r^2^ values ranged from 0.77 to 0.9 (Fig. 3b, Suppl. Table 3). Growth rates were 0.105±0.0035 mm ln(day)^−1^ for pH 8.0, 0.103±0.0017 mm ln(day)^−1^ for pH 7.6, and 0.079±0.0046 mm ln(day)^−1^ for pH 7.2. pH had a significant effect on larval growth (p > 0.001) and post-hoc test showed that growth rates were significantly different between the three pH treatments (p > 0.01). Replicate also had a significant effect on larval growth rate (p > 0.001) and a post-hoc test showed that at pH 7.6 growth rate in one of the replicates (number 3) was significant different from the other two and at pH 7.2 larval urchins in all three replicates had different growth rates.

## 

**Acidification induced overall shape change**

Based on the canonical variate analysis, larval urchins reared under the three pHs differed significantly in overall shapes (Fig. 4, p > 0.0001, Suppl. Table 4). Canonical variate 1 (CV1) and CV2 accounted for 65.4% and 20.7% of the total variance in the population. CV1 mainly varied with the ratio of body size and arm length; individuals with a lower CV1 scores had shorter total arm length but relatively longer arms per unit length of the body at a given size. CV2 varied with the arm lengths (overall height) and the separation distances between arms; individuals with a lower CV2 scores had shorter arms that are less spread out in the horizontal direction (Suppl. Table 4, Fig. 5). In general, larval urchins in the low pH treatments had a more negative CV2 score at a given CV1 score.

Our previous analysis showed that larval urchins had different growth rates when reared under different pH, therefore, we compared growth patterns of the three pH treatments by regressing the CV1 and CV2 scores against Procrutes centroid sizes. Regressions for CV1 were significant and had r^2^ value over 0.62 (Suppl. Table 6). Such allometric relationships did not hold for CV2 and the variance explained by the regression were low (r^2^ > 0.05). In other words, larvae that were larger in size did not necessary have longer overall height, arm lengths, or shorter separation distances between arms (CV2) (Suppl. Table 6).

## 

**Larval swimming in shear is not affected by pH**

Net movement and swimming metrics of larvae were computed after flow subtraction. For upward swimming larvae, average vertical swimming velocities differed between larval age and flow conditions (p > 0.0001, Suppl. Table 7, Fig. 5). At a given age, upward swimming larvae exposed to shear condition had higher vertical velocities. Maximum upward swimming speed was observed 10 days post fertilization and subsequently decreased with age. pH alone did not have a significant effect on vertical velocities (p = 0.67). There were also no significant interactions between pH and flow (p = 0.10) Net horizontal velocity was not affected by the three factors studied i.e., flow, age, and pH (p ≥ 0.12, Suppl. Table 7, Fig. 5).

For downward swimming larvae, average vertical and horizontal velocities differed between larval age and flow (p > 0.001, Suppl. Table 7, Fig. 6). Vertical velocities became less negative as larvae aged. Horizontal velocities were higher in shear than in still condition for larval urchins at a given age, i.e., there is more horizontal movement towards downward moving flow when exposed to shear. Larval urchins in all three pH treatments had higher horizontal velocities at 7 and 10 days post-fertilization. However, pH alone did not have a significant effect on vertical velocities (p = 0.24). There were also no significant interactions between pH and flow (p = 0.26, Suppl. Table 6, Fig. 6).

At a same day of age, larval urchins from the different pH treatments had different total body lengths. Such delay in growth could have potential implications for larval swimming (Fig. 3b). However, statistical comparison on the effect of flow and pH with size as a covariate could not be performed because there was very little overlap in total body length between the pH treatments when the video observations were performed. Larvae reared in pH 7.3 did not catch up in size over the 14 days of observations (Suppl. Fig. 2).

## Discussion and conclusion

Swimming of planktonic larvae has significant consequences for population dynamics and is tightly coupled with larval physiology and biomechanics^7^. When reared under decreased pH conditions, larval green urchins had reduced growth rates, changed overall shapes but were able to maintain their age-specific swimming performances.

### Larvae reared under decreased pH grew slower and in a different shape

Within the observed pH range (8.0–7.2), reduction in pH had no significant effect on larval survivorship over 14 days (Fig. 3a). This observation supports previous observation on the same species which identified pH 7.0 as the physiological tipping point^18^. The rate and pattern of growth were significantly impacted by pH (Fig. 3b). Reduced growth rate could prolong pelagic larval duration and reduce the number of settlers due to the high mortality in the plankton^10^,^19^,^20^. These slower growing individuals might also settle at smaller sizes^21^ or less discriminately to the chemical cues^22^ which could have negative implications on juvenile survival in the wild.

In addition to differences in rate of growth, overall shapes of the acidified larval urchins also differed from those of the control (Fig. 4). Previous studies on pH impact on larval morphology focus on linear measurement and have shown contrasting results: in some observations, including that on larval green urchins, ratio between body length and arm length/stomach length were affected by pH^18^,^23^,^24^ but not in others^25^,^26^. In the latter cases, the pH effect became non-significant when correcting for the reduced growth rate by including body length as a covariate in the analysis. In our data set, CV1 scores correlated significantly with Procrustes centroid sizes such that the relative ratio between larval body and arms varied in an allometric manner. However, the lack of correlation between Procrutes centroid size and CV2 scores suggest that the change in length of arms and separation distances between them do not allometrically increase with overall size (Suppl. Table 6). The differences between different aspects of shape change (CV scores) relative to overall size may help reconcile the difference in previous reports.

Observed shape changes under decreased pH may have significant biomechanical implications for larval movement. Larvae with the lower CV1 and 2 scores appeared to have an overall more “squat” morphology. This decrease in arm extension without the corresponding change in body length (decrease in CV1 score) would increase the distance between larval centers of buoyancy and gravity, and hence, the torque exerted when tilted in moving water would be decreased, thereby conferring stability^7^,^27^. In addition, the reduction in separation distance between arms (decrease in CV score) present a narrower width exposed to the shear gradient, which would result in less difference in water velocity across the larval body, less tilting of the larva^8^,^13^. Future modeling effort on fluid interactions of larvae should considered this kind of coordinated morphological variation in “armed larvae”.

### Decreased pH did not impact larval swimming

Contrary to the hypothesis that the change in shape would affect swimming, larval urchins raised under decreased pH had different age-specific morphology but maintained the same swimming performance in both still and moving water (Fig. 5, 6).

While pH had no significant effect on age-specific larval swimming velocities, this performance metric significantly varied with larval age. For example, older larvae had higher horizontal velocities and may be more likely entrained in downward moving waters by crossing flow lines. This could translate into differential transport through ontogeny^28^. It is remarkable that this age-specific pattern was maintained within our tested pH range despite significant differences in growth rates. This could be a consequence of the observed shape change allowing the smaller larvae raised in decreased pH to maintain stability. Other non-mutually exclusive mechanisms could also allow larval urchins to maintain their swimming performances despite changes in overall shape. First, larvae raised in decreased pH are smaller in size (this study) and could potentially be less calcified^29^. These morphological differences could in turn reduce the neutral weight of an individual, and hence, a relatively smaller lifting force is required to propel the larva^13^,^30^. Second, change in pH is reported to affect ciliary activities of the ciliate *Paramecium caudatum,* and a decrease in pH reduced the amount of ciliary reversals and sensitivity to KCl stimulation^31^. In sea urchins, ciliary bands play a key role in swimming and swimming speeds could be maintained through reducing number of reversal, such that more of the work done by the cilia would be directed to forward propulsion. Pluteus of other sea urchins (*Hemicentrotus pulcherrimus* and *Anthocidaris crassispina*) are able to swim at the same speed regardless of compass direction, implying a compensation for variation in gravitational pull^32^. Both this and our observation that plutei maintain their age-specific swimming abilities despite slower growth suggest that propulsive activities of larval urchins are under strong selective pressure, actively modulated, and highly plastic.

### Ecological implications for plasticity in swimming behaviors

Larval urchins use their ciliary bands for both food capture and swimming suggesting potential tradeoff between the two functions^8^. Volume of water filtered per unit time and hence food capture is dependent on total length of the ciliated band and its leakiness. Changes in larval size, shape, and weight may affect particle capture^33^. Also, if ciliary reversal is affected by changes in pH, particle capture rate would also decrease^34^. However, previous incubation experiment showed increased feeding rates in green urchin plutei raised under decreased pH^35^. This was interpreted as a compensatory mechanism following a decrease in digestion efficiency.

Maintaining age-specific swimming performance under ocean acidification could also have implications for larval transport. Earlier modeling and observation on pluteus larvae of sand dollars suggested that the changes in morphology and swimming through ontogeny affect selective transport such that older larvae were less stable (higher horizontal velocity in shear) and are more likely to be transported downwards^28^. This prediction also matches vertical distribution of larval sand dollars in the field; older larvae are found in deeper waters^36^. If this is the case, the observed maintenance of age-specific swimming behavior regardless of developmental stage implies larvae raised under decreased pH might be transported to deeper water prematurely, e.g. before being able to settle (competency). This mismatch between behavior and development could potentially decouple competent window from approaching of settlement sites.

In conclusion, larval *S. droebachiensis* demonstrated strong phenotypic plasticity in the face of decreased pH. The changes in overall shape did not negatively impact on larval swimming ability in still and moving water. On the contrary, this shape change to a more “squat form” might have helped provide stability. Maintained performance of swimming under OA condition reinforces the importance of this ecological function to early life stages. While larval urchins demonstrated capacity to cope with decreased pH in the lab, it is important for future studies to evaluate the ecological consequences of these functional impacts acting in concert. Together, these small, sub-lethal changes could negatively impact survivorship and transport, and ultimately the distribution and abundance of populations. However, this observation also illustrates the importance to include relevant functional parameters as endpoint and to avoid over-interpretation of *change* (e.g. in size) as negative consequences.

## Methods

### Larval rearing and seawater chemistry

Adult sea urchins *Strongylocentrotus droebachiensis* were collected from Droebak, Norway. Two males and two females were spawned and gametes were fertilized with protocol previously described. Larvae were kept at 10 individuals mL^−1^ at 9°C and fed a constant diet of 150 µg C L^−1^ *Rhodomonas* sp. (~ 6000 cells mL^−1^) starting five days post fertilization when their mouths opened. Three pH treatments (pH_T_ 8.0, 7.6, and 7.2) were used, with three replicate jars for each pH level. These pH levels correspond to average surface ocean today (8.0), predicted average value for 2100 and present-day extreme (7.6), and extreme prediction for 2100 (7.2)^18^. pH levels were maintained through CO_2_ bubbling and monitored with pH electrode and alkalinity titration^18^.

### Larval mortality and growth

**Figure 1:**
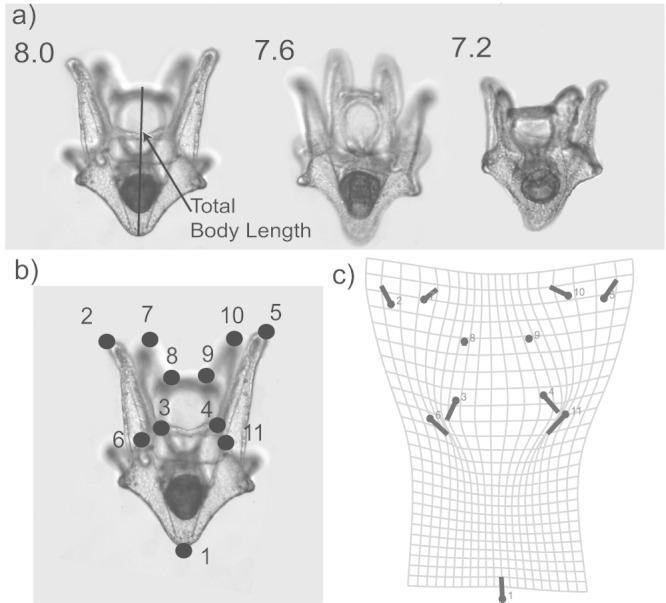
Micrographs of larval *Strongylocentrotus droebachiensis* (10 days post fertilization) reared at three nominal pH levels (a). Geometric morphometric techniques were used to analyze shape change in these larvae through identification of landmarks (b), followed by computation and comparison of Procrutes coordinates on transformation grids (c).

**Figure 3:**
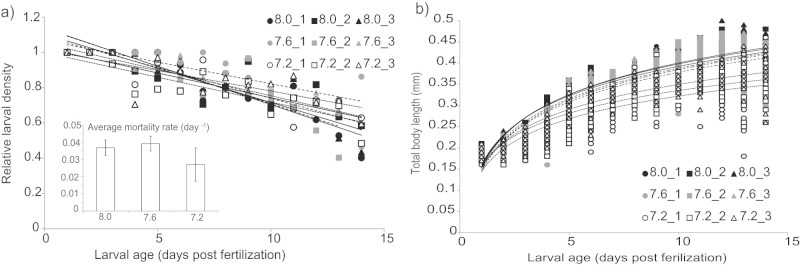
Average relative mortality rate (inset of a) calculated from linear regression of the change in larval density over time did not differ between pH treatments (a). However, pH has a significant effect on larval growth rate computed by logarithmic regression of increase in total body length over time (b). Closed symbols and solid block line represent pH 8.0 replicates, gray symbols and dash line represent pH 7.6, and open symbols and dotted line represent pH 7.2.

**Figure 4:**
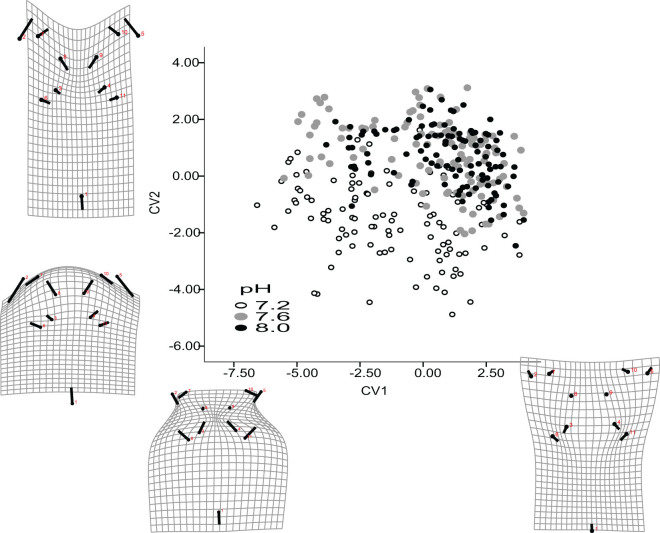
Larval urchins reared at different pHs had significantly different overall shape after accounting for size difference using landmark analysis. The transformation grids render the space between the landmarks with thin plate splines to aid visualization. On these grids each lollipop shows the original starting point of a landmark with a filled circle and the shift of landmark to the target shape is indicated by a line. Each dot represents the canonical variate scores of an individual observed in each of the pH treatments, color of the dots indicate different pH treatment. CV1 and CV2 together account for over 85% of the total variance observed and their transformation grids with the corresponding transformation grids are shown as insets.

**Figure 5:**
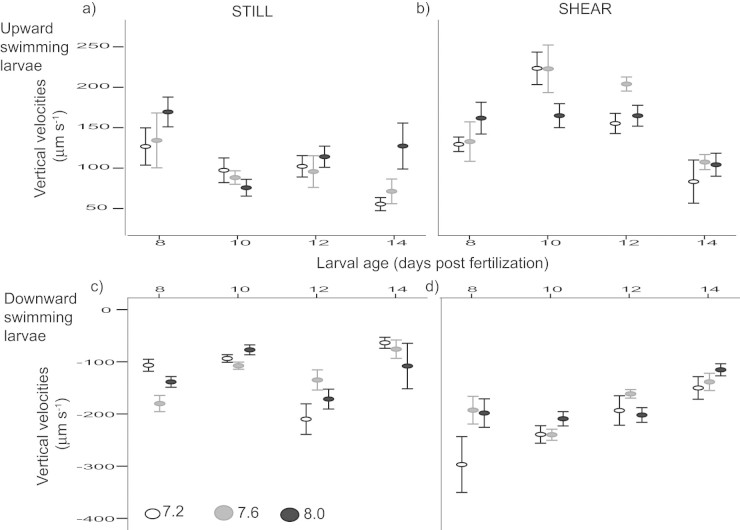
Vertical velocities of both upward and downward swimming larval urchins varied with age and background flow conditions but pH had no effect on larval swimming. Averages and standard errors of larval swimming velocities from each pH treatment are shown.

**Figure 6:**
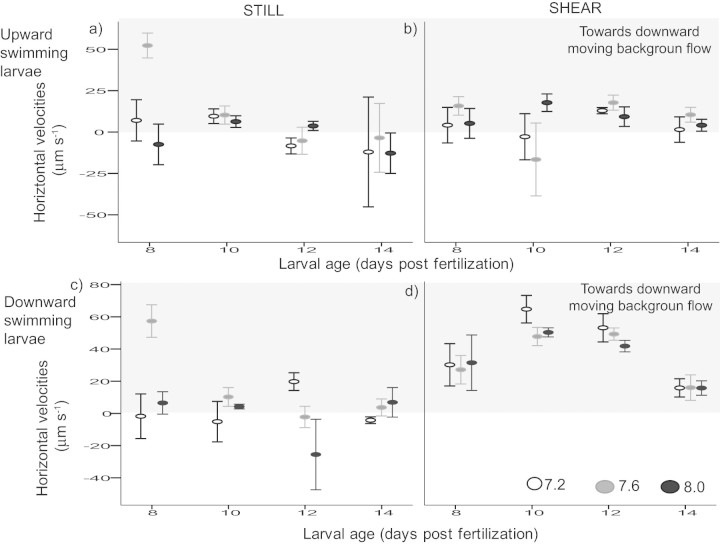
Horizontal velocities (Average ± S.E.) of downward swimming larval urchins varied with age and background flow conditions (c,d). Downward swimming individuals (d) had higher horizontal velocities than upward swimming individuals (b) in shear i.e., larvae were crossing flow lines towards downward moving background flow (grey area). For both groups of larvae, pH had no effect on larval horizontal swimming velocities.

Duplicate 10 mL subsamples were taken and fixed from each replicate daily. The number of larvae was counted under a dissecting microscope and the change in relative density was used to calculate mortality rate through linear regression against time. Micrographs of twenty individuals were taken and the total body lengths (Fig. 1) were measured at different times. A logarithmic regression i.e. change in body length (mm) per the natural log of unit time (days) was used to compute the growth rate (mm ln(day)^−1^).

### Geometric morphometrics analysis

Eleven landmarks were identified including tip of the larval body, tips and bases of posterodorsal arm and preoral arms (Fig. 1). These landmarks were digitized for 10 randomly selected individuals from each replicate at four time points 4, 7, 10, and 13 days post fertilization using the imaging software Fiji^37^. The coordinates extracted were imported into the software MorphoJ for General Procrustes Analysis^38^. To reduce the dimensionality of the data set, a canonical variate analysis was performed and changes in overall shape were visualized with transformation grids.

### Video motion analysis

Swimming behaviors were observed at four different time points (4, 7, 10, and 13 days post fertilization). Larvae were observed under no induced flow (still) and in moving water with a linear vertical velocity gradient (shear). Shear is a realistic simplification of turbulence in the natural environment for small larvae, as argued in previous studies^28^. The same shear tank from these studies was used to create shear by enclosing a narrow inner chamber with two flanking outer chambers held at different temperatures (Suppl. Fig. 1). Observations were made at the larval rearing pH and the two flow conditions were compared: in still condition where the temperature of both outer chambers were held at 10°C, and in shear where the temperature of the outer chambers were 0 and 20°C respectively. The temperature in the middle of the inner chamber was therefore held close to the rearing temperature at 10°C (+/− 1°C) and checked at the beginning of each observation.

Trajectories of larval urchins (200 larvae per trial) and those of algal tracer particles (~6000 cells mL^−1^) were recorded using a modified HD webcam (Logitech Pro Cam 7200) equipped with a CCTV lens at 7.5 fps under fiber optic lights illumination. Two, five minutes long video clips were first collected in still water and two additional clips in shear after a ten minute acclimation time. Video was processed and analyzed using Avidemux2.4 and Tracker3D^15^,^39^. Smoothing splines were applied to each trajectory to remove frame rate noise and to differentiate the overall direction of travel (axis) from the helical path (path). Swimming speed was computed based on the path, net vertical and horizontal velocity based on the axis. Flow subtraction is applied to all velocities calculation because the observed larval movement is the sum of both the passive transport by the moving water and the individual swimming behavior^40^. The background flow was computed by binning the algal trajectories from 1500 frames into 13 × 17 spatial bins, and the flow velocity in each bin was the median of all the trajectories passing through each bin. The 20°C temperature difference used in the observation chamber resulted in shear ranging from −0.6 to 0.25 s^−1^. Given the horizontal velocity generated was negligible, only the vertical component of the larval speed was corrected. Data were divided into up-swimming and down-swimming individuals based on overall displacement for statistical analysis, such that up-swimmers are those with their final positions above their starting position (i.e. a positive change z-coordinates) and down-swimmers are those with the final positions below their starting position (i.e. a negative change in z-coordinates). In this current scheme, the down-swimmers include both individuals that are sinking through ciliary arrest and those that are actively swimming downward through ciliary reversal.

### Statistical Analysis

The statistical analyses were performed using SAS or PASW software. Statistical assumption of normality and homogeneity of variance were tested with Shapiro-Wilk test and Levene's test. For ANCOVAs, additional test of homogeneity of regression slope and linearity in relation between covariate and dependent variable were performed with scattered plot and linear regressions. pH and replicate effect on carbonate chemistry were tested using an ANOVA. pH and replicate effect on mortality and survival were tested using an ANCOVA with age as covariate. pH effect on shape (CV scores) was tested using an ANOVA and the scores were also linearly regressed against centroid sizes. Effect of pH, flow, and their interactions on larval swimming was tested using an ANCOVA with age as covariate. Post-hoc Tukey's tests were performed where appropriate. All significant levels were set at p > 0.05.

## Author Contributions

K.C. and S.D. conceived and designed the experiment. K.C., E.G., S.D. conducted the experiment. K.C. and S.D. analyzed the data and prepared the manuscript.

## Supplementary Material

Supplementary Information Supplementary Information

## Figures and Tables

**Figure 2:**
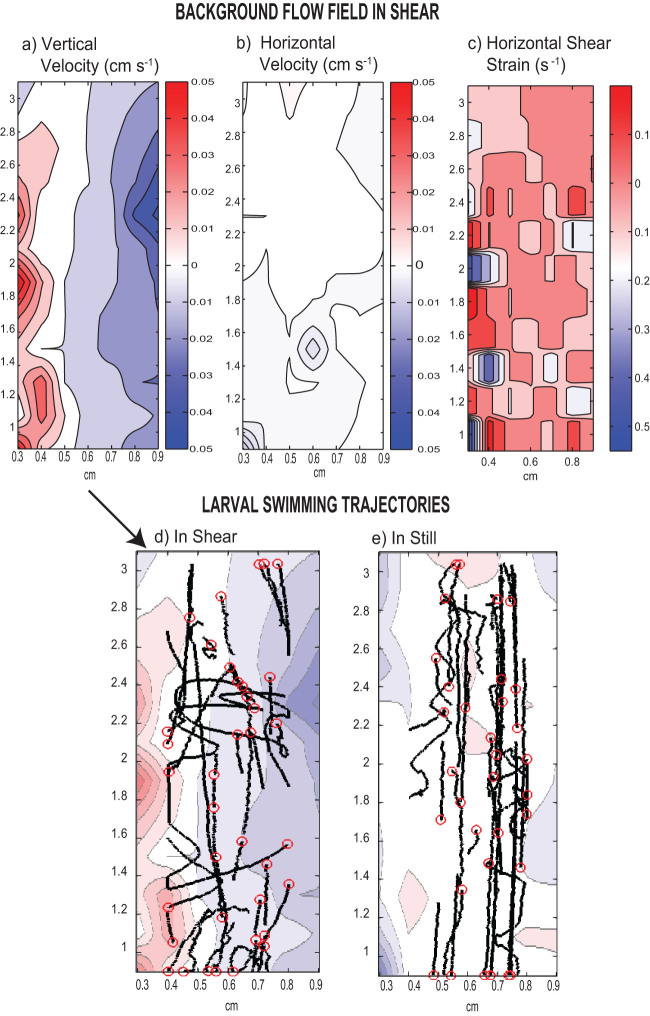
Example of background flow field in shear and swimming behaviors observed in two flow conditions (still and shear). Shear refers to a velocity gradient in the horizontal direction (a, b) and the shear level generated is similar to that in coastal water c). Red indicates upward moving water and blue indicates downward moving water. Swimming trajectories observed over five minutes of larvae reared at pH_T_ 8.0 were overlaid on these flow fields, with the red circles indicating the beginning of the paths (d, e). Under shear condition, larval urchins experienced horizontal transport across flow lines.
