# Supplementary material for: Acidification reduced growth rate but not swimming speed of larval sea urchins
Source: Sci Rep. 2015 May 15;5:9764. doi: 10.1038/srep09764 (PMC4432868; doi:10.1038/srep09764)

**Acidification reduced growth rate but not swimming speed of larval sea urchins**

Kit Yu Karen Chan^1, 2,*^, Eliseba García^3^, Sam Dupont^4^

^1^ Biology & Applied Ocean Physics and Engineering, Woods Hole Oceanographic Institution, Woods Hole, MA, USA

^2^ Division of Life Science, Hong Kong University of Science and Technology, Clear Water Bay, Hong Kong

^3^ Departamento de Biología Animal (Ciencias Marinas), Universidad de La Laguna, La Laguna, Tenerife, Islas Canarias, Spain

^4^ Department of Biological and Environmental Sciences, University of Gothenburg, The Sven Lovén Centre for Marine Sciences – Kristineberg, Fiskebäckskil, Sweden.

* Corresponding author:

Tel: +852 2358-7998

Email: [karenchan@ust.hk](mailto:karenchan@ust.hk)

Supplementary Table 1. Carbonate chemistry of the all replicates in the three experimental treatments. Total scale pH and total alkalinity (mean ± standard error) were measured and the remaining parameters were computed using CO2SYS.

|  |  | Measured | | | | | | Calculated | | | | | | | | |
| --- | --- | --- | --- | --- | --- | --- | --- | --- | --- | --- | --- | --- | --- | --- | --- | --- |
| Nominal pH | | pH tot | | | TA(µmol kg-1) | | | pCO2 (µatm) | | | Ωca | | | Ωar | | |
| 8.00 | 1 | 8.04 | ± | .02 | 2253.0 | ± | 1.4 | 397.5 | ± | 19.2 | 2.89 | ± | .13 | 1.82 | ± | .08 |
|  | 2 | 8.03 | ± | .01 | 2247.0 | ± | 41.0 | 413.0 | ± | 15.7 | 2.78 | ± | .01 | 1.76 | ± | .01 |
|  | 3 | 8.06 | ± | .04 | 2257.5 | ± | 20.5 | 384.3 | ± | 46.5 | 3.09 | ± | .17 | 1.96 | ± | .11 |
|  | Mean | 8.04 | ± | .03 | 2252.5 | ± | 21.0 | 398.3 | ± | 26.9 | 2.92 | ± | .17 | 1.84 | ± | .11 |
| 7.60 | 1 | 7.56 | ± | .02 | 2278.0 | ± | 2.8 | 1344.8 | ± | 60.7 | 1.06 | ± | .03 | .67 | ± | .02 |
|  | 2 | 7.76 | ± | .03 | 2267.0 | ± | 24.0 | 831.4 | ± | 79.2 | 1.60 | ± | .08 | 1.01 | ± | .06 |
|  | 3 | 7.74 | ± | .11 | 2257.0 | ± | 29.7 | 889.4 | ± | 260.3 | 1.54 | ± | .35 | .98 | ± | .22 |
|  | Mean | 7.68 | ± | .11 | 2267.3 | ± | 19.5 | 1021.9 | ± | 280.7 | 1.40 | ± | .31 | .88 | ± | .20 |
| 7.20 | 1 | 7.33 | ± | .07 | 2282.0 | ± | 22.6 | 2363.5 | ± | 356.5 | .65 | ± | .08 | .41 | ± | .05 |
|  | 2 | 7.21 | ± | .02 | 2286.0 | ± | 17.0 | 3123.7 | ± | 457.2 | .49 | ± | .06 | .31 | ± | .03 |
|  | 3 | 7.09 | ± | .06 | 2277.0 | ± | 24.0 | 4115.1 | ± | 104.7 | .38 | ± | .02 | .24 | ± | .02 |
|  | Mean | 7.21 | ± | .11 | 2281.7 | ± | 17.1 | 3200.8 | ± | 828.6 | .50 | ± | .13 | .32 | ± | .08 |

Supplementary Table 2. Linear regressions between time and relative larval density (RLD) in each replicate.

| pH | Replicate | Regression | r^2^ | F | p |
| --- | --- | --- | --- | --- | --- |
| 8.0 | 1 | RLD = -0.043 x Time + 1.14 | 0.85 | 69.9 | <0.0001 |
|  | 2 | RLD = -0.028 x Time + 1.02 | 0.69 | 26.2 | <0.0001 |
|  | 3 | RLD = -0.039 x Time + 1.10 | 0.80 | 49.4 | <0.0001 |
| 7.6 | 1 | RLD = -0.025 x Time + 1.07 | 0.70 | 27.5 | <0.0001 |
|  | 2 | RLD = -0.043 x Time + 1.13 | 0.73 | 30.2 | <0.0001 |
|  | 3 | RLD = -0.035 x Time + 1.09 | 0.85 | 65.8 | <0.0001 |
| 7.2 | 1 | RLD = -0.029 x Time + 1.05 | 0.76 | 38.6 | <0.0001 |
|  | 2 | RLD = -0.028 x Time + 1.00 | 0.71 | 28.8 | <0.0001 |
|  | 3 | RLD = -0.023 x Time + 1-01 | 0.59 | 17.1 | <0.0001 |

Supplementary Table 3. Logarithmic regression between larval total body length (BL) and time in each replicate.

| pH | Replicate | Regression | r^2^ | F | p |
| --- | --- | --- | --- | --- | --- |
| 8.0 | 1 | BL = 0.105 x ln(Time) + 0.157 | 0.90 | 2469.9 | <0.0001 |
|  | 2 | BL = 0.108 x ln(Time) + 0.153 | 0.87 | 1915.4 | <0.0001 |
|  | 3 | BL = 0.101 x ln(Time) + 0.161 | 0.90 | 2436.0 | <0.0001 |
| 7.6 | 1 | BL = 0.101 x ln(Time) + 0.145 | 0.83 | 1325.8 | <0.0001 |
|  | 2 | BL = 0.104 x ln(Time) + 0.145 | 0.84 | 1376.1 | <0.0001 |
|  | 3 | BL = 0.104 x ln(Time) + 0.149 | 0.83 | 1381.2 | <0.0001 |
| 7.2 | 1 | BL = 0.077 x ln(Time) + 0.144 | 0.68 | 594.0 | <0.0001 |
|  | 2 | BL = 0.077 x ln(Time) + 0.158 | 0.77 | 957.1 | <0.0001 |
|  | 3 | BL = 0.085 x ln(Time) + 0.154 | 0.80 | 1128.8 | <0.0001 |

Supplementary Table 4. Eigen values, population variance, and canonical structure coefficients for canonical variates analysis performed on landmark data for larval urchins using pH treatments as identifying grouping variables.

| CV | Eigenvalues | | % Variance | | Cumulative % | |  |
| --- | --- | --- | --- | --- | --- | --- | --- |
| 1 | | 5.25911 | | 65.448 | | 65.448 | |
| 2 | | 1.663879 | | 20.706 | | 86.154 | |
| 3 | | 0.346085 | | 4.307 | | 90.461 | |
|  | |  | |  | |  | |
|  | | Canonical Coefficients | | | | | |
| Landmark  Coordinate | | CV1 | | CV2 | | CV3 | |
| x1 | | -6.5317 | | 3.5056 | | 11.4542 | |
| y1 | | -9.2817 | | 13.1445 | | 5.9462 | |
| x2 | | -6.7941 | | 8.8955 | | -13.746 | |
| y2 | | -3.3142 | | -3.3707 | | 5.0874 | |
| x3 | | 0.0323 | | -9.0231 | | 16.5002 | |
| y3 | | 10.7426 | | -7.9146 | | -20.1883 | |
| x4 | | 10.5517 | | 1.5007 | | -9.7952 | |
| y4 | | 1.3439 | | 7.6529 | | 4.6974 | |
| x5 | | 5.0178 | | -7.0835 | | 7.113 | |
| y5 | | -5.7214 | | -2.3601 | | 6.392 | |
| x6 | | 10.5371 | | 7.4196 | | -2.1541 | |
| y6 | | 6.9462 | | -2.9664 | | 0.5933 | |
| x7 | | 4.6655 | | 5.1227 | | 20.2262 | |
| y7 | | -4.0673 | | -1.5763 | | -2.9653 | |
| x8 | | -8.0708 | | 3.5053 | | -16.3764 | |
| y8 | | 3.403 | | 3.3765 | | -12.4471 | |
| x9 | | 8.7088 | | -4.8202 | | 6.0345 | |
| y9 | | -11.9257 | | 1.8659 | | 16.2743 | |
| x10 | | -10.5288 | | -3.8966 | | -8.3874 | |
| y10 | | 1.9103 | | -2.6638 | | -7.0126 | |
| x11 | | -7.5878 | | -5.126 | | -10.869 | |
| y11 | | 9.9643 | | -5.1878 | | 3.6228 | |

Supplementary Table 5. Overall shape of larval urchin estimated by canonical variate scores (CV1 and CV2) varied significantly with age and pH treatments based on an ANCOVA with age as covariate and pH as fixed factors.

|  |  | Df | MS | F | P |
| --- | --- | --- | --- | --- | --- |
| Age | CV1 | 1 | 1244.583 | 853.6132 | <0.0001 |
|  | CV2 | 1 | 115.0085 | 101.4518 | <0.0001 |
| pH | CV1 | 2 | 128.4495 | 88.0987 | <0.0001 |
|  | CV2 | 2 | 189.708 | 167.346 | <0.0001 |
| Error | CV1 | 329 | 1.458018 |  |  |
|  | CV2 | 329 | 1.133628 |  |  |

Supplementary Table 6. Linear regression for CV scored against Procrutes centroid sizes.

|  | pH | Regression | R2 | F | p |
| --- | --- | --- | --- | --- | --- |
| CV1 | 8.1 | CV1 = 0.060 x Centroid-10.86 | 0.788 | 407.9 | <0.0001 |
|  | 7.7 | CV1 = 0.008 x Centroid-13.66 | 0.722 | 291.0 | <0.0001 |
|  | 7.3 | CV1 = 0.010 x Centroid-16.33 | 0.622 | 205.4 | <0.0001 |
| CV2 | 8.1 | CV2 = -0.001 x Centroid+2.647 | 0.077 | 9.139 | 0.003 |
|  | 7.7 | CV2 = -0.001x Centroid+3.088 | 0.077 | 9.407 | 0.003 |
|  | 7.3 | CV2 = -0.001 x Centroid+4.30 | 0.037 | 3.995 | 0.048 |

Supplementary Table 7. Swimming velocities (vertical, w and horizontal, u) of larval urchins varied with age and flow conditions but not pH according to an ANCOVA with age as covariate, pH and flow as fixed factors. * indicates significant difference with p value smaller than 0.05.

|  |  | Upward swimming | | | | | Downward swimming | | | | |
| --- | --- | --- | --- | --- | --- | --- | --- | --- | --- | --- | --- |
|  |  | df | MS | F | p |  | df | MS | F | p |  |
| Age | u | 1 | 1543.10 | 2.43 | 0.12 |  | 1 | 5222.72 | 6.76 | 0.01 | * |
|  | w | 1 | 34412.21 | 12.91 | <0.0001 | * | 1 | 59169.30 | 15.57 | <0.0001 | * |
| pH | u | 2 | 441.89 | 0.70 | 0.50 |  | 2 | 1305.98 | 1.69 | 0.19 |  |
|  | w | 2 | 1058.08 | 0.40 | 0.67 |  | 2 | 5507.86 | 1.45 | 0.24 |  |
| Flow | u | 1 | 729.59 | 1.15 | 0.29 |  | 1 | 36657.65 | 47.41 | <0.0001 | * |
|  | w | 1 | 86971.13 | 32.63 | <0.0001 | * | 1 | 168340.56 | 44.30 | <0.0001 | * |
| Flow x pH | u | 2 | 556.69 | 0.88 | 0.42 |  | 2 | 1768.98 | 2.29 | 0.11 |  |
|  | w | 2 | 6147.77 | 2.31 | 0.10 |  | 2 | 5123.93 | 1.35 | 0.26 |  |
| Error | u | 128 | 635.74 |  |  |  | 134 | 773.15 |  |  |  |
|  | w | 128 | 2665.72 |  |  |  | 134 | 3799.78 |  |  |  |

Supplementary Figure 1. Vertical shear (velocity gradient δu/δz) was generated in experimental observation chamber through temperature differential.


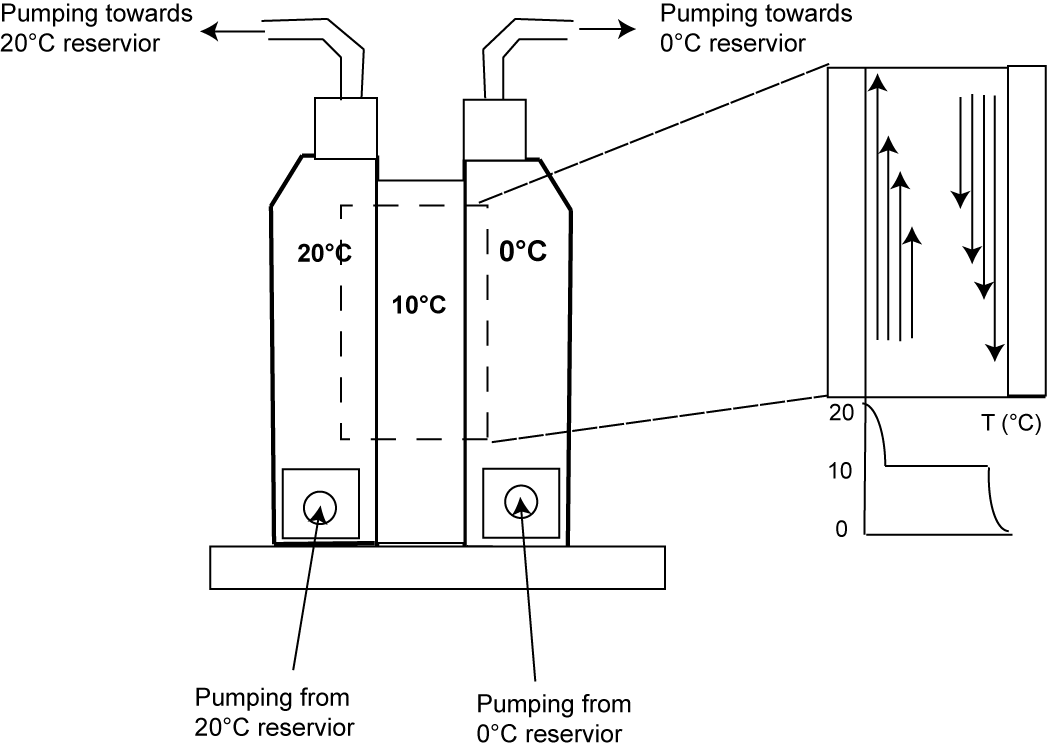


Supplementary Figure 2. Swimming velocities (mean ± standard error) of larval urchin in still water and in shear plotted again calculated total body length.


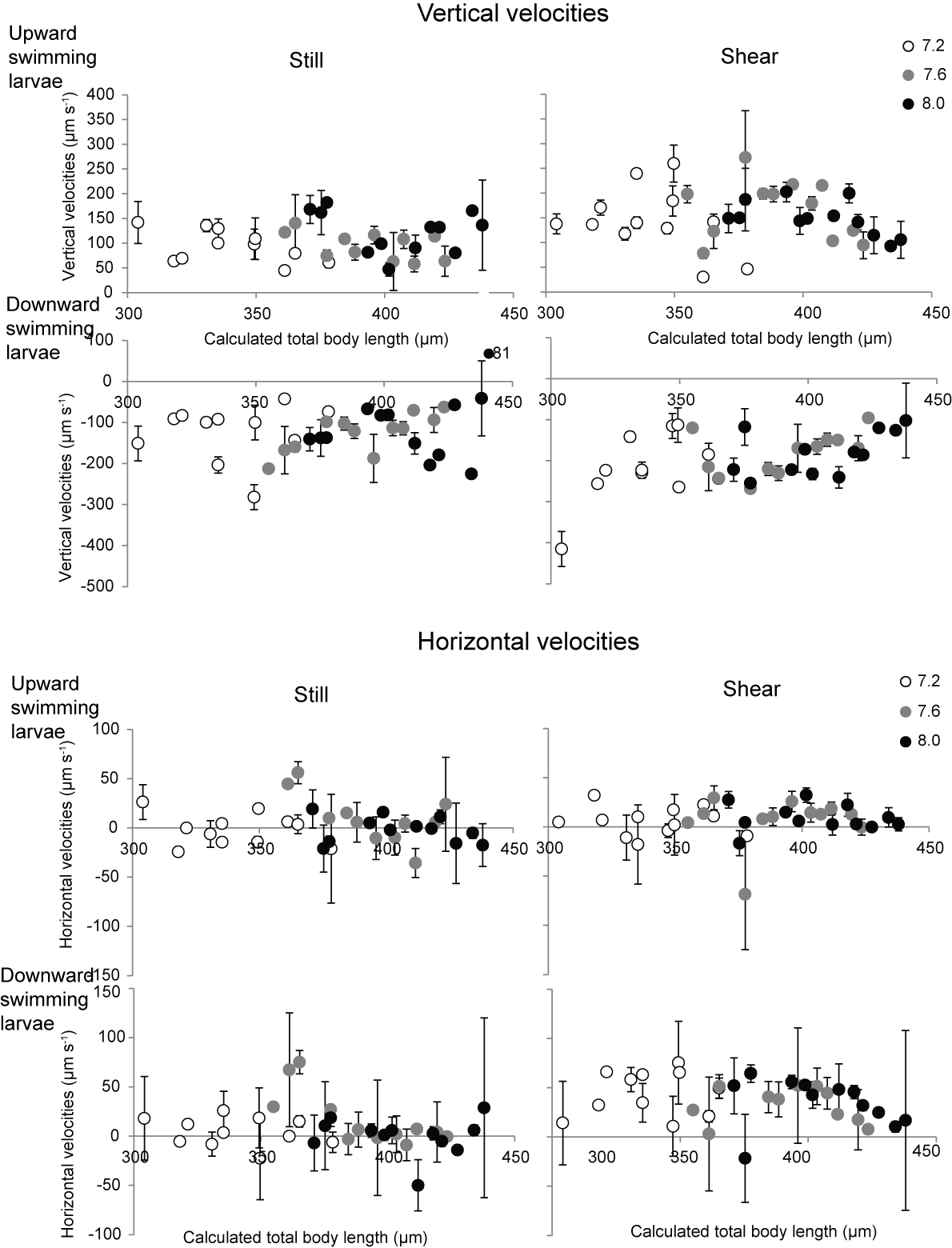

Supplement: Supplementary Information [file srep09764-s1.doc]
